# Co-evolution of resistance and virulence in *Klebsiella pneumoniae* liver abscess: PLA-specific mechanisms and therapeutic dilemmas

**DOI:** 10.3389/fcimb.2026.1767477

**Published:** 2026-02-10

**Authors:** Han Lin, Zhenghaoyu Huang, Yonghong Guo

**Affiliations:** 1 School of Gongli Hospital Medical Technology, University of Shanghai for Science and Technology, Shanghai, China; 2 Department of Infectious Disease, Gongli Hospital of Shanghai Pudong New Area, Shanghai, China

**Keywords:** antimicrobial resistance, carbapenem resistance, hypervirulent *Klebsiella pneumoniae*, liver abscess, plasmid fusion, virulence factors

## Abstract

The co-evolution of resistance and virulence in *Klebsiella pneumoniae* poses a significant challenge in the management of pyogenic liver abscesses (PLA), particularly with the advent of carbapenem-resistant hypervirulent *K. pneumoniae* (CR-hv*KP*). This review specifically addresses PLA to consolidate current knowledge on how key virulence factors—such as the K1/K2 capsule, hypermucoviscosity, and aerobactin—contribute to hepatic infection. It also examines the molecular mechanisms, including plasmid fusion and horizontal gene transfer, that are believed to facilitate the convergence of hypervirulence and carbapenem resistance. Additionally, the review discusses the unique clinical challenges presented by CR-hv*KP* in the context of PLA, including diagnostic delays, antimicrobial treatment failures, and complications in drainage. Emerging countermeasures, such as rapid molecular diagnostics and novel anti-virulence strategies, are also explored. By integrating contemporary molecular insights with the specific clinical challenges of PLA management, this review provides an updated translational perspective aimed at bridging the gap between pathogenesis and therapeutic strategies for CR-hv*KP*-associated infections.

## 1 Introduction

Hypervirulent *Klebsiella pneumoniae* (hv*KP*) is a globally disseminated pathogen strongly associated with severe invasive infections, particularly pyogenic liver abscess (PLA). Initially recognized as a geographically restricted pathogen within the Pacific Rim during the 1980s, hv*KP* has now become a significant global public health concern, with cases reported in 52 countries spanning five continents (Liu et al., 1986). In contrast to classical *Klebsiella pneumoniae* (cKP), hv*KP* is capable of causing primary PLA in immunocompetent individuals and is linked to a high risk of distant metastatic dissemination.

In endemic regions, PLA is the hallmark manifestation of hv*KP*, accounting for > 50% (up to 90.9%) of community-acquired cases (Ye et al., 2016; Angeles-Solano et al., 2025).A retrospective study of 26 pediatric PLA cases in Senegal found *K. pneumoniae* in 7.7% of isolates, with a hospital prevalence of 100 cases per 100,000 admissions (Ba et al., 2016). Genomic surveillance indicates that its intercontinental spread is primarily facilitated by six major “super clones” (e.g., clonal group [CG]23, CG258), with 33.3% of strains currently co-harboring carbapenemase resistance genes (Jiang et al., 2025; Nagendra et al., 2025a). This phenomenon represents a distinct evolutionary pathway, termed “virulence-resistance co-evolution”—which is mediated by insertion sequences (IS*5*, IS*Kox3*) that promote capsular phase variation and enable plasmid fusion and horizontal gene transfer (Wei et al., 2025).

Carbapenem use exerts selective pressure, facilitating convergence of hv*KP* and carbapenem-resistant *K. pneumoniae* (CR*KP*) pathways, resulting in the emergence of a dual-threat pathogen: carbapenem-resistant hypervirulent *Klebsiella pneumoniae* (CR-hv*KP*) (Du et al., 2020). Following the emergence of resistance, mortality rates increase significantly, with CR-hv*KP* exhibiting a pooled mortality rate of 57%, nearly three times higher than the 21% observed for hv*KP*, and presenting over a 12-fold increased odds of mortality compared to carbapenem-susceptible hv*KP* (Nagendra et al., 2025c). This pathogen attains its dual threat through the integration of virulence plasmids (e.g., pLVPK-like) and resistance plasmids harboring carbapenemase genes (e.g., *bla*_KPC-2_). Such integration may result in the formation of hybrid plasmids, potentially facilitating the evolution of non-K1/K2 strains into CR-hv*KP* (Chen et al., 2023). Infections caused by CR-hv*KP* are linked to adverse clinical outcomes; for instance, bloodstream infections have a mortality odds ratio (OR) of 4.05, with a pooled in-hospital mortality OR of 2.46 (Chen et al., 2025). Involvement of the central nervous system is associated with a fatality rate of 48.5%–53%, which approaches 100% in cases of CR-hv*KP*-related intracranial infections (Xu et al., 2019). Within the predominant Asian clone ST11 infections, in-hospital mortality is reported at 33.5%, while CR-hv*KP*-specific mortality ranges from 33% to 42% (Zhao et al., 2023; Wang et al., 2024). Furthermore, CR-hv*KP*-associated liver abscesses exhibit a twofold increase in treatment failure and mortality rates compared to those caused by classical *Klebsiella pneumoniae* strains.

Traditional paradigms positing a virulence-resistance trade-off are no longer applicable to CR-hv*KP*. The pathogenesis of CR-hv*KP* is shaped by three primary challenges: (1) accelerated tissue destruction and immune evasion, driven by increased capsule production and enhanced iron acquisition systems; (2) a marked reduction in therapeutic options due to carbapenem resistance; and (3) antibiotic tolerance arising from the unique abscess microenvironment, which impedes bacterial eradication despite adequate drug concentrations (Wang et al., 2022). A comprehensive understanding of the molecular interactions between resistance determinants and virulence factors—particularly the dynamics of plasmid fusion, mechanisms of horizontal gene transfer, and the reconfiguration of transcriptional regulatory networks—is crucial for addressing this therapeutic challenge. Such insights will form the foundation for developing targeted prevention strategies and novel antimicrobial agents against this emerging superbug. **Table 1** provides a summary of the key molecular and clinical distinctions between classical hypervirulent *Klebsiella pneumoniae* (hv*KP*) and emerging CR-hv*KP* clones, offering a comparative framework for the subsequent discussion.

**Table 1:** Comparison of hv*KP* and CR-hv*KP* liver abscesses: from molecular characteristics to clinical outcomes.

Category	Subtype/key type	Source and predominant st types	Core definition and characteristics	Major virulence determinants	Resistance profile/susceptibility	Clinical features and complications	Mortality
hv*KP* (non-CR)	K1/K2 Serotypes	Primary Source: Community-acquired infections, especially in the Asia-Pacific region. Predominant STs: ST23 (K1), ST65 (K2), ST86, etc.	Causes primary liver abscesses, susceptible to most antibiotics (except ampicillin). Exhibits a hypermucoviscous phenotype and is prone to hematogenous dissemination.	pLVPK-like virulence plasmid carrying *rmpA/A2* (mucoid phenotype), *iucABCD/iutA* (aerobactin), *iroBCDN* (salmochelin). K1/K2 capsular serotype.	Typically does not carry ESBL or carbapenemase genes. Susceptible to carbapenems, third-generation cephalosporins, etc.	High-risk hosts: Extremely high proportion of diabetic patients (~50-60%). Typically presents as community-acquired PLA (often single, large abscess), with high propensity for metastatic spread, particularly endophthalmitis and meningitis (Fang et al., 2007; Sohrabi et al., 2022).	Relatively low But can rise sharply due to complications. Uncomplicated liver abscess: With timely drainage and effective antibiotics, mortality is typically <10% (Lee et al., 2017; Chan et al., 2022). With metastatic infection: e.g., purulent meningitis, mortality can reach 20-30% or higher (Tang et al., 2023).
CR-hv*KP*	ST11-KL64 Clone (Current Major Epidemic Clone)	Primary Source: Reported in both hospital and community settings, with increasing prevalence in China and globally. Predominant ST: ST11, often associated with KL64 capsular type.	Combines the broad-spectrum resistance/high transmission efficiency of ST11 with the invasiveness of hv*KP*. A major threat for nosocomial infections.	Acquires *rmpA, iuc, iro* genes via acquisition of pLVPK-like virulence plasmids or hybrid plasmids carrying virulence gene clusters. Capsular types are mostly KL47, KL64.	Produces KPC-2 carbapenemase (*bla*_KPC-2_), often co-harbors other resistance genes (e.g., *rmtB*), conferring resistance to multiple antibiotics (Wang et al., 2024).	Primarily causes severe nosocomial infections (pneumonia, bloodstream infection, sepsis). Can also cause liver abscess and metastatic infections. Disease progression is rapid, with high risk of progression to septic shock (Wei et al., 2022).	Extremely high. Multiple studies report mortality rates of 40-60%, identified as an independent risk factor for death (Chen et al., 2021; Wu et al., 2021).
	Emerging ST23 Type (Hypervirulent Clone Acquiring Resistance)	Source: Complex, reported in both hospital and community settings, constituting a new major threat. Predominant ST: ST23. Predominant Serotype: K1.	The classic K1/ST23 hv*KP* strain evolves into CR-hv*KP* by acquiring resistance plasmids. Retains all hypervirulent traits of the ST23 clone (e.g., hypermucoviscous phenotype, high invasiveness) while gaining extensive drug resistance, posing a significant potential hazard.	Carries the complete pLVPK-like virulence plasmid; highly virulent, harboring all key virulence genes (iuc, iro, *rmpA/A2*). K1 capsule.	Acquired resistance via acquisition of plasmids carrying carbapenemase genes (e.g., *bla*_NDM-1_, *bla*_KPC-2_, *bla*_OXA-48_) through conjugation or other mechanisms. May form a complex genomic structure co-harboring the large virulence plasmid and multiple resistance plasmids (Tu et al., 2024).	Clinical presentation is similar to classic ST23-K1, still characterized by liver abscess and high-risk metastatic infections (eye, brain). However, treatment is extremely difficult due to resistance, leading to a dramatically increased risk of infection control failure and complication worsening.	Extremely high. Due to severely limited treatment options and strong bacterial virulence, some studies report mortality >50% (Zhou et al., 2021).
	Other Emerging Threat Clones (e.g., ST15, ST147)	Evolved from internationally prevalent high-risk MDR/XDR clones (ST15, ST147) acquiring virulence plasmids.	Converged clones exhibiting extreme drug resistance (XDR/PDR) and hypervirulence. ST147 is closely associated with the global spread of NDM-1.	Acquires virulence gene clusters via plasmids; carriage of specific virulence factors varies by clone and plasmid.	Harbors multiple resistance mechanisms, particularly various carbapenemases (KPC, NDM, OXA-48, etc.), often exhibiting extensive drug resistance (XDR) or pan-drug resistance (PDR) (Davoudabadi et al., 2022; Zhao et al., 2022).	Often cause complex infections. Due to the near absence of effective therapeutic options, there is an extremely high risk of uncontrollable systemic dissemination and sepsis, leading to critical illness.	High. Mortality is similar to that of the ST11 clone, with extremely limited treatment choices, resulting in similarly high mortality rates.

hv*KP*, hypervirulent *Klebsiella pneumoniae*; CR-hv*KP*, carbapenem-resistant hypervirulent *K. pneumoniae*; ST, sequence type; CPS, capsular polysaccharide; MDR, multidrug-resistant; XDR, extensively drug-resistant; PDR, pan-drug-resistant; ESBL, extended-spectrum beta-lactamase.

While existing reviews have extensively addressed hypervirulent *Klebsiella pneumoniae* (hv*KP*) or carbapenem-resistant *Klebsiella pneumoniae* (CR*KP*), this article offers a unique perspective centered on PLA. It synthesizes the most recent evidence on the co-evolution of CR-hv*KP* to develop a comprehensive analysis that spans from molecular mechanisms to clinical implications for this life-threatening infection. The objectives of this review are as follows: first, to consolidate current knowledge on the fundamental virulence mechanisms (e.g., K1/K2 capsule, hypermucoviscosity, aerobactin) that specifically drive the pathogenesis of liver abscesses; second, to elucidate the molecular mechanisms, particularly plasmid fusion and horizontal gene transfer, that contribute to the concerning convergence of hypervirulence and carbapenem resistance; third, to examine the resulting clinical and therapeutic challenges unique to the management of CR-hv*KP* liver abscesses, including diagnostic delays, antimicrobial treatment failures, and difficulties in drainage; and finally, to explore emerging countermeasures, ranging from rapid molecular diagnostics to novel anti-virulence strategies and prevention approaches informed by co-evolutionary dynamics. This review synthesizes recent molecular epidemiological findings with the practical challenges of clinical management to provide a comprehensive and updated perspective that aims to connect mechanistic understanding with effective therapeutic strategies for CR-hv*KP*-associated PLA.

## 2 Pathogenesis: from intestinal colonization to liver abscess formation

The development of primary liver abscess caused by hv*KP* begins with intestinal colonization, proceeds via hematogenous dissemination, ultimately leading to liver abscess formation. The intestine acts as a reservoir and potential source for both hv*KP* and its drug-resistant variants. Asymptomatic intestinal colonization is a necessary precursor for subsequent endogenous infections (Gorrie et al., 2017). Colonization may commence through the interaction of bacterial fimbriae with extracellular matrix proteins on host enterocytes, facilitating adherence. The bacteria employ capsular polysaccharides to form biofilm-like structures that resist clearance by intestinal peristalsis, bile salts, and digestive enzymes (Martin and Bachman, 2018; Roqunuzzaman et al., 2025). During this phase, hv*KP* must contend with the gut commensal microbiota and evade host immune defenses. Its robust siderophore systems, including aerobactin and salmochelin, exhibit a high affinity for iron, surpassing that of host transport proteins, thus facilitating the bacterium’s establishment of a dominant colonization density (Wang et al., 2025). The administration of broad-spectrum antibiotics disrupts the homeostasis of the gut microbiota and diminishes competitive inhibition against *K. pneumonia*, thereby conferring a colonization advantage to these strains (Calderon-Gonzalez et al., 2023).

In scenarios of gut dysbiosis or compromised host immunity, hv*KP* is capable of translocating across the intestinal mucosal barrier and entering the portal venous system, leading to hematogenous dissemination (Lin et al., 2014; You et al., 2024). Although Kupffer cells constitute the liver’s intrinsic immune defense, hv*KP* can inhibit the nuclear translocation of the pivotal transcription factor NF-κB, thereby attenuating early inflammatory responses and immune cell recruitment (Hoh et al., 2019). Upon establishment in the liver, the bacteria exploit their advanced siderophore systems to acquire iron, facilitating rapid proliferation. Simultaneously, the hypermucoviscous phenotype, regulated by virulence plasmid-encoded factors such as RmpA, enhances bacterial aggregation and imparts anti-phagocytic properties, thereby promoting the development of micro-abscesses. This bacterial proliferation induces significant local inflammation, resulting in hepatocyte necrosis and extensive neutrophil infiltration. Ultimately, the necrotic tissue, along with bacteria, inflammatory cells, and fibrin, becomes encapsulated, leading to the formation of a characteristic PLA.

## 3 Mechanisms of principal virulence factors in liver abscess formation

### 3.1 Capsular polysaccharide and colonization advantage of serotypes K1/K2

The capsular polysaccharide of *Klebsiella pneumoniae* constitute a substantial, gel-like layer of acidic polysaccharides that envelops the bacterium’s outermost surface. This structure is instrumental not only for serological typing but also as a crucial mechanism for evading host immune detection and facilitating infection. Among the over 80 identified capsular serotypes, K1 and K2 are most closely linked with hypervirulent phenotypes and invasive disease, particularly prevalent in patients with liver abscesses (Zhang et al., 2019). The pathogenic efficacy of hv*KP* in inducing liver abscesses is largely attributable to the synergistic interactions between its capsular polysaccharide (CPS) and the K1/K2 serotypes.

The primary pathogenic mechanism of CPS resides in its robust capacity to undermine host immunity, primarily through two complementary processes: resistance to phagocytosis and inhibition of complement activity. CPS obstructs the deposition of C3 fragments on the bacterial surface, thereby diminishing complement-mediated opsonization and phagocytosis (Moranta et al., 2010). This hydrophilic polysaccharide gel effectively conceals pathogen-associated molecular patterns (PAMPs) on the bacterial outer membrane, thereby impeding recognition and binding by host phagocytes. Neutrophils and macrophages, which constitute the primary line of immune surveillance, are directly obstructed by these mechanisms (Ke et al., 2025). Furthermore, CPS actively disrupts the activation of the complement system, a vital component of the host’s innate immunity. Upon activation, opsonin molecules such as complement C3b are produced, which coat pathogen surfaces to facilitate opsonophagocytosis. The distinctive chemical structure of the *KP* capsule effectively inhibits the activation of the classical, alternative, and lectin complement pathways (Xu et al., 2024a). By means of steric hindrance, it prevents the formation and stabilization of C3 convertase on the bacterial surface, significantly reducing C3b deposition. Kupffer cells serve as an essential defensive barrier against hematogenous pathogens. However, K1/K2 strains substantially enhance their survival and virulence within the liver by evading capture and destruction by Kupffer cells. This evasion ultimately leads to localized inflammatory responses and purulent accumulation, resulting in the formation of liver abscesses (Huang et al., 2022).This process is essential for the initial hepatic colonization by hv*KP*, facilitating bacterial survival in the bloodstream, rapid proliferation within hepatic tissue, and subsequent extensive—albeit often ineffective—neutrophil infiltration.

Patients with diabetes mellitus, particularly type 2 diabetes, constitute the highest-risk population for *KP* liver abscesses (Gu et al., 2025). A 2016–2017 study of 163 *KP*-PLA cases reported that 49.7% of patients had diabetes mellitus, 30.7% had hypermucoviscous strains, and 40.5% had K1 serotype (Zhang et al., 2019). In addition to compromised immune functions, such as neutrophil dysfunction, the metabolic disturbances inherent in diabetes create conditions conducive to bacterial virulence. Hyperglycemic environments have been associated with increased capsular polysaccharide (CPS) production. High glucose concentrations upregulate the expression of capsular biosynthesis genes by reducing intracellular cyclic AMP (cAMP) levels and impairing the function of the cAMP-CRP complex, leading to a significant increase in capsule production (Lin et al., 2013; Chen et al., 2023). Capsular thickening undoubtedly augments bacterial resistance to phagocytosis, thereby exacerbating pathogenicity (Gu et al., 2025). In addition, intrahepatic mannose concentrations are significantly increased in diabetic patients, which may facilitate the synthesis or structural modification of K1/K2 capsules, thereby enhancing bacterial evasion from Kupffer cell capture (Dorman et al., 2018). Collectively, the K1/K2 serotypes represent a fundamental pathogenic mechanism in the formation of hv*KP* liver abscesses by inhibiting complement deposition, resisting macrophage phagocytosis, and modulating metabolism in the context of diabetes.

### 3.2 Hypermucoviscous phenotype and RmpA-mediated immune evasion

Beyond capsular serotypes, the hypermucoviscous (HMV) phenotype, driven by high-level capsular polysaccharide (CPS) production, serves as another hallmark of hv*KP* pathogenicity. Formation of this phenotype is tightly regulated by virulence regulators, most notably *rmpA* and *rmpA2*. As a positive regulator, RmpA activates the *rmpADC* operon and RmpC protein to stimulate expression of the CPS gene cluster, promoting capsule synthesis. Deletion of *rmpA* reduces capsule production, leading to capsule loss or thinning and a significant reduction in bacterial virulence and immune evasion capacity (Lin et al., 2019; Xu et al., 2024a). The regulatory network governing *rmpA* and *rmpA2* expression involves complex molecular mechanisms, including control by the fumarate and nitrate reduction regulatory protein (FNR). FNR promotes CPS production by regulating expression of the CPS gene cluster, thereby contributing to HMV phenotype establishment. This regulatory cascade not only enhances immune evasion but also facilitates persistent bacterial survival and dissemination within the host through physical barrier formation and immunosuppressive effects.

The HMV phenotype also suppresses host immune responses through multiple pathways. Capsular polysaccharide inhibits the release of neutrophil extracellular traps (NETs) via physical barrier effects and immune evasion mechanisms, reducing bacterial clearance. Concurrently, high-level CPS production and enhanced adhesive capacity promote bacterial persistence within abscesses, creating an “immune-privileged niche” that further impairs host immune clearance.

### 3.3 Siderophore system: aerobactin-mediated iron acquisition

Aerobactin serves as the principal iron acquisition system in hv*KP*, enabling efficient sequestration of free iron in the iron-restricted host microenvironment to satisfy requirements for rapid bacterial proliferation. The *IucA* enzyme encoded by the *iucA* gene synthesizes this citrate-hydroxamate siderophore, whose affinity far exceeds that of host-derived iron-binding protein lipocalin-2, maintaining efficient iron uptake in iron-limited host niches, thereby circumventing nutritional immunity and markedly enhancing pathogenicity (Russo et al., 2015). Studies demonstrate that nearly all hv*KP* strains harbor aerobactin synthesis genes (*iuc*), with a prevalence of 93%–100% in hv*KP* versus only ~6% in c*KP* (Jun, 2018).

Clinical epidemiological investigations have identified a significant association between aerobactin-positive *KP* strains and community-acquired liver abscess. A Singaporean multicenter study identified the co-presence of *rmpA*, mucoid phenotype, and aerobactin as critical factors underlying bacteremic liver abscess (Tan et al., 2019). Systematic reviews further confirmed that aerobactin synergizes with other virulence determinants (e.g., K1/K2 serotypes, *rmpA*, *magA*) to create a dual-defense mechanism of “iron acquisition plus mucoid protection,” enabling bacteria to evade immune clearance in the bloodstream and rapidly colonize hepatic tissue (Oka et al., 2025). In animal models, *KP* strains with *iuc* gene knockout exhibit significantly reduced lethality and hepatic tissue invasion capacity, indicating that aerobactin is an essential factor in liver abscess formation (Liu et al., 2022). Additionally, aerobactin genes are frequently located on large plasmids that concurrently harbor multidrug resistance genes (e.g., *bla*_KPC_*, bla*_NDM)_, posing a clinical challenge of virulence-resistance co-evolution. Recent cases of extended-spectrum β-lactamase-producing *KP* strains carrying iuc genes have emerged, suggesting that resistance and siderophore systems can co-disseminate via horizontal gene transfer, further compounding therapeutic dilemmas (De Francesco et al., 2023).

## 4 Molecular mechanisms and driving factors of resistance-virulence co-evolution

### 4.1 Fusion plasmids: vectors for co-transfer of resistance and virulence genes

The co-evolution of antimicrobial resistance and hypervirulence is closely linked to the dynamic recombination and horizontal transfer of genetic material, particularly mobile genetic elements (MGEs) such as plasmids. In hv*KP* strains, such as those belonging to capsular serotypes K1 or K2, the hypervirulent phenotype is predominantly determined by a large virulence plasmid (Chen et al., 2004). The most representative of these is pLVPK and its analogs, which typically exceed 200 kb and harbor a repertoire of critical virulence gene clusters (Yang et al., 2022), including *rmpA* and *rmpA2* genes encoding regulators of the mucoid phenotype, and gene clusters for siderophore systems such as aerobactin (Russo and Marr, 2019). These virulence plasmids commonly contain genes involved in CPS synthesis regulation; for instance, *rmpA* positively regulates capsular synthesis, enabling bacterial resistance to phagocytosis by host cells and complement-mediated killing—a crucial mechanism for immune evasion and development of invasive infections such as liver abscesses (Walker and Miller, 2020). However, these canonical virulence plasmids are typically non-conjugative, lacking the complete set of genes required for conjugative transfer (e.g., the tra gene cluster), thereby limiting their intrinsic horizontal transfer capacity between strains (Dai and Hu, 2022).

Acquisition of antimicrobial resistance primarily relies on resistance plasmids (R-plasmids). Unlike virulence plasmids, many resistance plasmids are conjugative and can efficiently transfer between different bacteria and even across species, representing the primary route for rapid dissemination of antimicrobial resistance (Sattler et al., 2024). The key to resistance-virulence co-evolution lies in the integration of resistance gene modules into virulence plasmids or the fusion of two distinct plasmids. Resistance genes are frequently inserted into the same plasmid or co-residing multi-replicon plasmids via transposons or integrons, forming “virulence-resistance gene clusters”. Tn*1546*, a composite transposon of the Tn*3* family, can insert at 5-bp direct repeat sites and carry resistance genes such as *vanA*, indicating its high plasticity for intergenic horizontal transfer (Arthur et al., 1993). In hv*KP*, insertion sequences such as IS26 and IS1216 frequently flank Tn*1546* or other resistance elements to form composite transposons, enabling capture of resistance genes onto pLVPK or other large plasmids through replicative transposition or homologous recombination (Sletvold et al., 2010).

**Figure 1:**
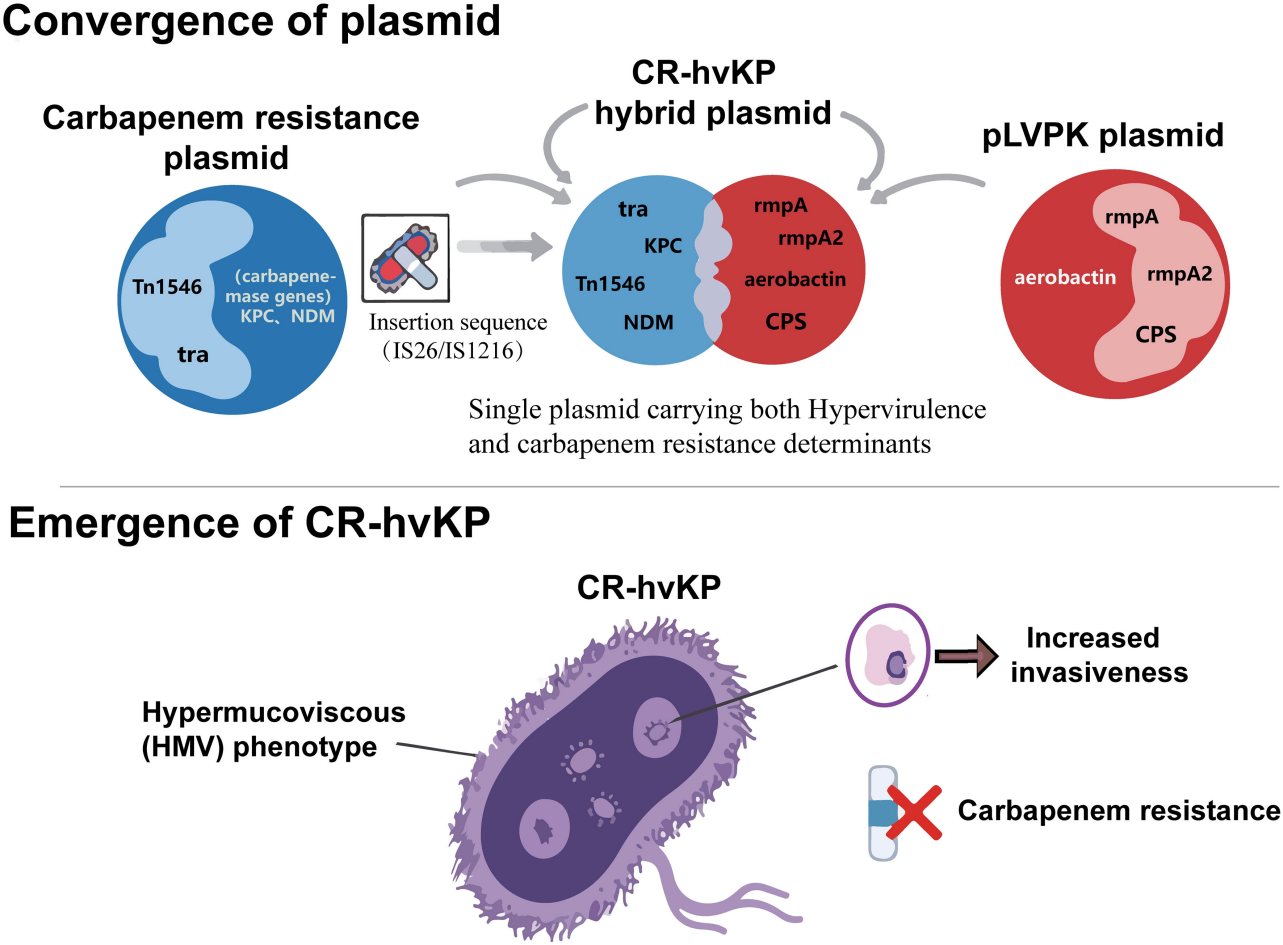
Schematic diagram of insertion sequence-mediated fusion of virulence and resistance plasmids and the formation of CR-hv*KP*.

When non-conjugative pLVPK carrying *rmpA*-family virulence genes coexist with conjugative plasmids harboring carbapenemase genes, homologous fusion sites within the cell facilitate homologous or single-strand exchange between the two plasmid types, generating hybrid plasmids. Such hybrid plasmids can simultaneously transfer both virulence and resistance phenotypes in a single conjugative event, markedly enhancing pathogenicity and drug tolerance in recipient bacteria. Genomic analyses have revealed that high-risk Klebsiella clones such as ST11 and ST15 often carry two independent plasmids: one non-conjugative pLVPK-like virulence plasmid and another self-transmissible MDR plasmid; these can form co-integrates through IS*26*-mediated composite transposition or homologous recombination, achieving “virulence-resistance co-transfer” (Dong et al., 2018). As illustrated in **Figure 1**, this insertion sequence-mediated fusion of virulence and resistance plasmids is a key molecular event in the formation of CR-hv*KP*. This plasmid-driven convergence directly translates into the severe clinical dilemmas discussed in the following section.

### 4.2 Driving role of antibiotic misuse

Inappropriate use of carbapenem antibiotics may confer a survival advantage to resistant strains. In clinical isolates of CR*KP*, resistance enzymes such as *bla*_kp-2_ and *bla*_NDM_ are frequently found in conjunction with hypervirulence genes, including *rmpA*, *iuc*, and *iro*, resulting in the formation of the CR-hv*KP* phenotype, as corroborated by multicenter epidemiological studies (Zhan et al., 2017). The two-component system (TCS) EnvZ/OmpR is integral to this process. EnvZ, a sensor kinase that detects changes in external osmotic pressure and pH, becomes activated under stress induced by carbapenems and subsequently phosphorylates OmpR. OmpR acts as a transcriptional regulator by binding to target promoters to modulate the expression of outer membrane porins OmpF and OmpC. Mutations or overexpression of OmpR can lead to decreased outer membrane permeability, thereby reducing the influx of carbapenems and enhancing resistance levels. Concurrently, OmpR directly upregulates several virulence genes, including those involved in siderophore systems and mucoid capsule synthesis, such as *rmpA* and *magA*, enabling the bacteria to simultaneously increase pathogenicity under antimicrobial pressure (Yuan et al., 2011).

Although direct evidence for carbapenem-induced upregulation of virulence genes in liver abscess isolates has not yet been fully established, transcriptomic studies offer supporting evidence from other infection model. Specifically, OmpR has been shown to directly regulate the expression of virulence determinants in pulmonary infections. The construction of ompR knockout strains has demonstrated that OmpR deficiency alters the bacterial gene expression profile during host cell infection, leading to the suppression of multiple key virulence factors and, consequently, attenuating bacterial pathogenicity (Janssen et al., 2024). Thus, the selective pressure exerted by antibiotic misuse perpetually drives the evolution and prevalence of CR-hv*KP*, enabling bacteria to simultaneously develop resistance and synergistically enhance their virulence. This dynamic interaction may contribute to a formidable combination of co-evolving resistance and virulence, significantly increasing the complexity and risk of treatment failure in clinical settings.

### 4.3 Quorum-sensing-mediated coordinated expression of resistance and virulence

In the resistance-virulence co-evolution mechanism of *Klebsiella pneumoniae*, the *luxS*/AI-2 quorum-sensing system mediates coordinated expression of multidrug resistance phenotypes and hypervirulence traits through bacterial density-dependent signal accumulation. The *luxS* gene in *K. pneumonia*e encodes AI-2 synthase, and its expression level positively correlates with AI-2 signal molecule accumulation, exhibiting constitutive expression characteristics (Zhu et al., 2011). Transcriptomic analyses have demonstrated that AI-2 activity peaks when *K. pneumonia*e enters the late logarithmic to early stationary growth phase, a process finely regulated by environmental factors including glucose concentration, pH, and boric acid (Zhu et al., 2012). When bacterial density reaches a threshold, AI-2 is internalized and phosphorylated via the Lsr operon transport system, activating downstream signaling cascades that induce global transcriptional reprogramming. AI-2 signaling activates downstream gene cluster expression through a phosphorylation cascade mediated by the two-component system LsrK/LsrR (Chen et al., 2020). This regulatory mode enhances serum resistance in hv*KP* while simultaneously reducing penetration efficiency of β-lactam antibiotics through the three-dimensional barrier effect of the biofilm matrix, thereby achieving “structural resistance.”

At the molecular mechanistic level, AI-2-LsrR binding may relieve transcriptional repression of downstream target genes by the Lsr transporter, thereby coordinately upregulating both resistance and virulence genes. Recent transcriptomic data demonstrate that eugenol treatment significantly reduces mRNA levels of *luxS*, *pfs*, *lsrR*, and *lsrK*, concomitant with biofilm dispersal and diminished resistance, indirectly supporting the functional association between AI-2 signaling pathways and resistance maintenance (Wang et al., 2019). Intervention experiments with quorum-sensing inhibitors (e.g., eugenol) have shown that AI-2 synthesis inhibition can reduce biofilm thickness by over 60% and restore susceptibility to carbapenems, indirectly confirming the necessity of quorum-sensing systems in resistance maintenance (Castillo-Juárez et al., 2015).

## 5 Therapeutic dilemmas of liver abscess caused by hypervirulent drug-resistant strains

### 5.1 Failure of conventional antimicrobial therapy and diagnostic delay

The molecular mechanisms described above directly translate into formidable clinical challenges in the management of CR-hv*KP* liver abscesses, as outlined below. Typical hv*KP* liver abscesses often appear on CT as a single, thin-walled, and regular abscess cavity containing necrotic material internally. Although this feature shows high specificity (98.6%) in differentiating hv*KP* from non-hv*KP* liver abscesses (Lee et al., 2011), its sensitivity is limited. In the early stages of infection, imaging may only reveal atypical diffuse hypodense lesions, which can easily lead to missed diagnosis or misinterpretation, and moreover, cannot indicate antibiotic resistance.

While conventional bacterial culture and antimicrobial susceptibility testing (AST) are considered the gold standard, they are time-consuming, typically requiring 48 to 72 hours to yield final results (Wenzler et al., 2023). During this period, clinicians are compelled to initiate empiric therapy, which carries a substantial risk of failure. Although the “string test” can serve as a rapid, simple method for identifying the hypermucoviscous phenotype, under multidrug resistance pressure, some hv*KP* strains may lose this classic trait, causing the test to yield false-negative results and potentially leading to misdiagnosis (Chang and Ong, 2022). The rapid progression of Hv*KP* liver abscess necessitates timely intervention, as treatment delays beyond 48 hours may result in missing the optimal intervention window, leading to irreversible organ damage and heightened mortality. Delayed appropriate therapy (DAT) has been definitively associated with a significantly increased risk of mortality in patients with severe infections (Lodise et al., 2018).

Although rapid diagnostic technologies, such as matrix-assisted laser desorption ionization-time of flight mass spectrometry (MALDI-TOF MS) and polymerase chain reaction (PCR), have emerged with the potential to reduce pathogen identification time to mere hours (Tsuchida et al., 2020), their widespread clinical adoption and standardization pose significant challenges. Furthermore, there is a paucity of large-scale prospective trial evidence demonstrating their impact on ultimate clinical outcomes, which hinders the complete replacement of conventional AST (Boattini et al., 2023).Diagnostic delays can hinder the initiation of treatment, and even when interventions are timely, their effectiveness is further compromised by the complexities associated with abscess management.

### 5.2 Challenges in antimicrobial treatment

Standard antimicrobial therapies are frequently ineffective against CR-hv*KP* liver abscesses. CR-hv*KP* strains frequently harbor multiple resistance genes, which impart resistance to a broad spectrum of first- and second-line antibiotics, including β-lactams, fluoroquinolones, and aminoglycosides, thereby significantly limiting the options available for empirical therapy (Mataseje et al., 2019; Feldman et al., 2022). As a result, treatment is often restricted to “last-line” agents such as colistin and tigecycline (Morrill et al., 2015). However, these agents present considerable limitations, including significant nephrotoxicity and poor tissue penetration, and they are associated with high rates of clinical failure when used as monotherapy (Xiao et al., 2018). Instances have been reported where CR-hv*KP* developed adaptive resistance during tigecycline treatment due to mutations in genes such as *ramR* and *lon*, leading to therapeutic failure (Jin et al., 2021).

In response to the limitations associated with monotherapy, combination therapy has emerged as an essential clinical strategy. Contemporary therapeutic approaches emphasize the development of novel β-lactam/β-lactamase inhibitor combinations and synergistic treatment modalities. Notably, agents such as ceftazidime/avibactam (CAZ-AVI) and meropenem/vaborbactam demonstrate significant efficacy against KPC-producing strains, thereby serving as pivotal options for the management of CR*KP* infections (Fang et al., 2023; Petrosillo et al., 2019). Furthermore, some case studies have reported the successful treatment of severe infections caused by CR-hv*KP* liver abscesses using a combination of meropenem-avibactam and gentamicin (Yang and Wang, 2024).

In addition to the challenges posed by antimicrobial resistance, the pathological architecture of liver abscesses presents pharmacokinetic/pharmacodynamic (PK/PD) obstacles. The thickened fibrous capsule functions as a physical barrier, significantly impeding drug diffusion into the abscess core, which often results in local drug concentrations that are insufficient to achieve the effective therapeutic threshold (Onufrak et al., 2016). The acidic, protein-rich, and hypoxic abscess microenvironment compromises antibiotic efficacy: acidity can destabilize aminoglycosides, while high protein content may bind drugs, reducing free concentrations. Furthermore, severe hypoxia diminishes bacterial metabolic activity, inducing a persistent state that markedly reduces the bactericidal effectiveness of β-lactams, which depend on active bacterial growth. Consequently, even when *in vitro* susceptibility testing indicates susceptibility, achieving both effective drug concentration and activity within the actual abscess environment remains challenging. Therefore, clinical strategies must prioritize drug penetration by extending infusion times for time-dependent antibiotics and ensuring adequate drainage to overcome the permeation barrier, thereby enhancing the likelihood of successful treatment.

### 5.3 Clinical limitations of abscess drainage combined with antimicrobial therapy

The management of hv*KP* liver abscess has reached a broad consensus over the past decades: effective abscess drainage is the key therapeutic strategy, primarily encompassing percutaneous catheter drainage (PCD) and surgical drainage (Ferraioli et al., 2008; Liu et al., 2013). Retrospective studies have demonstrated that patients undergoing percutaneous hepatic abscess drainage exhibit substantially higher clinical improvement rates compared with those without drainage (95.7% vs. 55.6%) and may experience reduced mortality risk (Li et al., 2023). While PCD has become the preferred approach in many clinical scenarios due to its minimally invasive nature and avoidance of general anesthesia, its application in hv*KP* is constrained by multiple factors. The pus in hv*KP*, composed of a large amount of capsular polysaccharide (CPS), bacteria, necrotic tissue debris, and inflammatory cells (such as neutrophils), exhibits extremely high viscosity. This pus offers substantial resistance during drainage and readily blocks the side holes and lumen of the catheter, leading to inadequate or complete drainage failure (Sato et al., 2022). The highly viscous pus readily obstructs small-bore catheters, necessitating larger-bore catheters with frequent irrigation, which increases procedural complexity and secondary infection risk. PCD failure rates are particularly elevated in multiloculated or large abscesses (>5 cm) (Serban et al., 2021), prompting consideration of surgical drainage for such cases due to potentially higher success rates and shorter hospital stays (Tan et al., 2005; Qian et al., 2016). Currently, high-quality prospective data remain lacking to provide evidence-based guidance on the superiority of PCD versus surgical drainage for hv*KP* liver abscess, particularly in patient subgroups with K1/K2 serotypes or concomitant risk factors such as diabetes mellitus.

### 5.4 Management challenges in specific patient populations

Particular attention is required for managing specific patient groups, such as diabetic patients, who are the primary susceptible population for hv*KP* liver abscesses (Liu et al., 2013; Xu et al., 2024b). Hyperglycemia significantly compromises neutrophil function, including chemotaxis, phagocytosis, and bactericidal activity, thereby diminishing the host’s capacity to eliminate *K. pneumonia* (Lin et al., 2021). Elevated blood glucose levels can enhance the expression of virulence factors, such as capsular polysaccharide and fimbriae, facilitating bacterial evasion of immune responses and the establishment of deep-seated infections (Nagendra et al., 2025b). Research has demonstrated that diabetic patients are at a substantially increased risk of developing distant metastatic infections, such as endophthalmitis and central nervous system infections, compared to non-diabetic individuals (Lin et al., 2006). Therefore, in addition to infection management and drainage, rigorous glycemic control is imperative.

Beyond individuals with diabetes, immunocompromised patients represent another high-risk group for hv*KP* infections (Liu et al., 2023). Impairment of neutrophil function significantly diminishes host clearance capacity, thereby creating favorable conditions for rapid bacterial proliferation and dissemination. For solid organ transplant recipients (SOTRs), patients with hematologic malignancies, or individuals undergoing long-term immunosuppressive therapy, both cellular and humoral immune functions are compromised (Dan et al., 2014). CR*KP* infections in SOTRs can lead to exceptionally high 30-day all-cause mortality rates, ranging from 18% to 71% (Clancy et al., 2013; Dan et al., 2014). Compared to immunocompetent patients, immunosuppressed hosts infected with CR-hv*KP* experience worse outcomes, characterized by higher treatment failure rates, increased incidence of septic shock, and greater mortality (Yang et al., 2020). Currently, the understanding of treatment strategies for CR-hv*KP* liver abscesses primarily stems from case reports and small-scale retrospective studies, with a notable absence of prospective trials focusing on immunocompromised populations (Tumbarello et al., 2012). These patients experience significantly elevated rates of intensive care unit admissions and mortality, and their bacterial isolates frequently exhibit multidrug resistance, thereby further constraining the already limited therapeutic options available.

## 6 Countermeasures and research prospects

### 6.1 Development and application of novel diagnostic technologies

The development and implementation of novel molecular diagnostic technologies capable of rapidly and simultaneously identifying pathogens, virulence phenotypes, and resistance genes has become an urgent imperative to combat CR-hv*KP* infections and improve patient outcomes. In recent years, PCR-based nucleic acid amplification technologies have achieved substantial progress, gradually emerging as essential tools for emergency infection diagnosis. Compared with conventional culture-based methods requiring several days, PCR technology can significantly reduce turnaround time for detection of pathogens and their genetic characteristics to within hours, sensitively detecting *KP*-specific genes and key genetic markers that determine hypervirulent phenotypes and resistance profiles (Fursova et al., 2021). The siderophore system genes (e.g., *iucA*) and capsular polysaccharide regulatory genes (e.g., *rmpA/rmpA2*) encoded on hypervirulence plasmids represent defining genetic signatures of hv*KP* that are strongly associated with invasive infections such as liver abscesses (Lee et al., 2016). Therefore, incorporating these genes into PCR panels can facilitate early identification of CR-hv*KP* in clinical practice, enabling initiation of optimal targeted or combination therapeutic regimens.

Beyond PCR technology, loop-mediated isothermal amplification (LAMP) demonstrates enormous potential for point-of-care testing (POCT) due to advantages including independence from sophisticated thermal cyclers, rapid reaction kinetics, and ease of result interpretation (Notomi et al., 2000). LAMP and recombinase polymerase amplification (RPA) technologies have been successfully applied for the rapid detection of *KP* virulence and resistance genes (Dong et al., 2015). Integration with lateral flow assay strips for visual result interpretation eliminates the need for expensive, complex instrumentation, substantially enhancing accessibility of rapid diagnostics. Although these isothermal amplification technologies may face challenges of false positives from non-specific amplification, diagnostic accuracy can potentially be improved through optimization of reaction systems and incorporation of novel gene editing tools such as CRISPR-Cas for specific recognition (Qiu et al., 2022).

### 6.2 Targeted anti-virulence therapies (bacteriophage/monoclonal antibodies)

The escalating crisis of antibiotic resistance has rendered the development of novel therapeutic agents with alternative antimicrobial mechanisms an urgent priority for hv*KP* treatment. The prominent virulence phenotype of hv*KP* provides ideal targets for “anti-virulence therapy.” Such strategies aim to disarm pathogens by inhibiting or neutralizing bacterial virulence factors rather than directly killing bacteria, thereby rendering them more susceptible to host immune clearance while theoretically reducing resistance selection pressure (Ali et al., 2021). Bacteriophage therapy and monoclonal antibodies represent the two most promising approaches in this field.

Bacteriophages, as natural predators of bacteria, are being reconsidered for their therapeutic potential in the era of antibiotic resistance (Iszatt et al., 2021). For hv*KP* strains causing liver abscesses, bacteriophage therapy offers unique advantages. Bacteriophages exhibit high host specificity, enabling precise targeting and lysis of pathogenic bacteria without disrupting the human commensal microbiota (Gholizadeh et al., 2024). In multiple animal models of hv*KP* infection, bacteriophage intervention has significantly reduced bacterial burden, improved host survival, and effectively controlled progression of liver abscesses and sepsis (Hung et al., 2011). However, clinical application of bacteriophage therapy remains in its infancy, with no large-scale, standardized Phase I/II clinical trial results available for hv*KP* liver abscess patients.

Similar to bacteriophage therapy, monoclonal antibodies (mAbs) provide a novel approach for precisely targeting hv*KP* virulence factors (Wang et al., 2016). Among numerous hv*KP* virulence factors, CPS represents the most extensively studied target for monoclonal antibody development due to its central role in immune evasion and anti-phagocytosis (Hung et al., 2011). Specific monoclonal antibodies targeting CPS of prevalent serotypes such as K1 (e.g., 4C5 and 19A10) have demonstrated efficacy in promoting opsonophagocytic clearance and controlling bacterial dissemination, substantially improving animal survival in murine infection models (Adamo and Margarit, 2018). Nevertheless, clinical translation faces challenges, including limited coverage of single antibodies due to capsular serotype diversity and undefined optimal dosing regimens and therapeutic windows in humans (Li et al., 2024).

### 6.3 Prevention strategies based on co-evolution mechanisms

Given the limitations of existing therapies, prevention strategies rooted in an understanding of co-evolution mechanisms are urgently needed. The co-evolution of antimicrobial resistance and hypervirulence has substantially compressed therapeutic options and significantly worsened patient prognosis, with mortality markedly elevated in patients with invasive infections caused by CR-hv*KP* (Garcia et al., 2021). Consequently, developing prevention strategies based on co-evolution mechanisms has become imperative. This endeavor encompasses not only traditional infection control measures but also requires intervention at the molecular level to disrupt coordinated expression and transmission of resistance and virulence genes.

Antimicrobial stewardship (AMS) constitutes the cornerstone for delaying resistance development. In confronting the challenge of resistance-virulence co-evolution, AMS programs require enhanced precision. However, for strains harboring virulence-resistance fusion plasmids, simply reducing use of specific drug classes may be insufficient to halt their dissemination. Future AMS strategies should integrate real-time genomic surveillance data to help clinicians predict the emergence and outbreak trends of high-risk clones, thereby enabling more proactive interventions.

The limitations of antibiotic therapy are becoming increasingly apparent, making the development of novel non-antibiotic interventions, particularly vaccines, a fundamental strategy to address the CR-hv*KP* threat. Current vaccine development strategies focus primarily on two directions: (1) developing multivalent CPS conjugate vaccines targeting predominant circulating serotypes (e.g., K1 and K2) (Lin et al., 2022); and (2) screening for highly conserved protein antigens across different clonal complexes (e.g., prevalent ST11 and ST23) such as outer membrane proteins OmpA and OmpK36 as targets for subunit or multi-epitope vaccines using reverse vaccinology approaches (Zhu et al., 2021). The ultimate goal is to induce production of specific antibodies that neutralize virulence factors and promote phagocytic clearance, thereby blocking disease progression at early infection stages and effectively circumventing bacterial resistance mechanisms to address the formidable challenge posed by resistance-virulence co-evolution.

## 7 Limitations

This review is subject to several limitations. As a narrative review, there is an inherent risk of bias in the selection and interpretation of literature. The focus is predominantly on liver abscesses, thereby not thoroughly addressing the characteristics of CR-hv*KP* at other infection sites. The existing clinical studies demonstrate significant heterogeneity in terms of definitions, methodologies, and patient populations, which impairs the comparability of the data. The current therapeutic evidence is predominantly derived from retrospective studies, with a notable lack of prospective, high-quality evidence. This is especially pertinent for specific populations, such as immunocompromised patients, and for emerging therapies like bacteriophages, where the evidence base is even more limited. Additionally, the perspective of this article is largely informed by the epidemiology of the Asia-Pacific region, which may restrict the global applicability of its conclusions. Recognizing these limitations is essential for an objective interpretation of this work and aids in identifying directions for future research.

## 8 Discussion

The convergence of hypervirulence and carbapenem resistance in *Klebsiella pneumoniae* presents a significant challenge, driven by molecular mechanisms such as insertion sequences IS*5* and IS*Kox3*. These sequences enable adaptive processes like capsular phase variation and plasmid fusion, facilitating the spread of virulence and resistance genes. Evidence highlights the role of hypervirulence factors, with the aerobactin synthesis gene *iuc* present in nearly all hv*KP* strains. Genetic, genomic, and animal model research confirms the importance of K1/K2 capsular serotypes, the hypermucoviscous phenotype, and hybrid plasmids in promoting co-evolution via horizontal gene transfer.

Clinically, in high-prevalence areas, suspected liver abscess cases should undergo PCR testing for virulence genes (*iucA*, *rmpA*) and carbapenemase genes (*bla*_KPC_, *bla*_NDM_) alongside traditional culture methods. In the management of infections, it is advisable to avoid monotherapy with tigecycline or colistin, and instead prioritize combination regimens that incorporate novel β-lactam/β-lactamase inhibitors, such as ceftazidime/avibactam. For abscesses exceeding 5 cm, those that are multiloculated, or those associated with K1/K2 serotypes, early assessment for drainage is imperative. Additionally, strict glycemic control and intensified, closely monitored therapy are vital for immunocompromised patients.

Nonetheless, these recommendations are not yet substantiated by robust high-level evidence. There is a notable absence of prospective trials that specifically compare drainage modalities or validate optimal antimicrobial combinations for CR-hv*KP* liver abscesses, with current guidelines primarily based on observational data. Addressing this dual threat necessitates a coordinated global strategy, which should include enhanced real-time genomic surveillance to monitor high-risk clones such as ST11, alongside sustained investment in research and development to expedite the creation of novel antimicrobials and alternative therapies, including bacteriophages and vaccines. Future research must aim to fill these critical evidence gaps. This entails the establishment of integrated clinical-genomic databases to enhance empirical therapy and elucidate epidemiological trends, the execution of prospective multicenter studies to compare treatment and drainage strategies within defined patient cohorts, and the acceleration of novel antivirulence therapies targeting capsule or siderophore systems, including monoclonal antibodies and bacteriophages, to offer innovative approaches for managing these challenging infections.
